# Comparative Genome Sequence Analyses of Geographic Samples of *Aspergillus fumigatus*—Relevance for Amphotericin B Resistance

**DOI:** 10.3390/microorganisms8111673

**Published:** 2020-10-28

**Authors:** Yuying Fan, Yue Wang, Jianping Xu

**Affiliations:** Department of Biology, McMaster University, Hamilton, ON L8S 4K1, Canada; fany8@mcmaster.ca (Y.F.); wangy660@mcmaster.ca (Y.W.)

**Keywords:** *Aspergillus*, amphotericin B, antifungal drug, resistance, *Aspergillus fumigatus*, whole-genome sequencing, comparative genomics

## Abstract

Amphotericin B (AMB) is a major fungicidal polyene agent that has a broad spectrum of action against invasive fungal infections. AMB is typically used as the last-line drug against serious and life-threatening infections when other drugs have failed to eliminate the fungal pathogens. Recently, AMB resistance in *Aspergillus fumigatus* has become more evident. For example, a high rate of AMB resistance (96%) was noted in the *A. fumigatus* population in Hamilton, Ontario, Canada. AMB-resistant strains have also been found in other countries. However, the mechanism of AMB resistance remains largely unknown. Here, we investigated the potential genes and mutations associated with AMB resistance using whole-genome sequences and examined AMB resistance distribution among genetic populations. A total of 196 whole-genome sequences representing strains from 11 countries were examined. Analyses of single nucleotide polymorphisms (SNPs) at the whole-genome level revealed that these strains belonged to three divergent genetic clusters, with the majority (90%) of AMB resistant strains located in one of the three clusters, Cluster 2. Our analyses identified over 60 SNPs significantly associated with AMB resistance. Together, these SNPs represent promising candidates from which to investigate the putative molecular mechanisms of AMB resistance and for their potential use in developing rapid diagnostic markers for clinical screening of AMB resistance in *A. fumigatus*.

## 1. Introduction

*Aspergillus fumigatus* is a globally distributed saprophytic mold that plays a major role in recycling environmental carbon and nitrogen. Its primary ecological niche is decaying organic matter, but it is also commonly found in the air, water and soil. The fungus has an abundant asexual reproduction cycle and produces a prolific number of asexual spores, known as conidia [1]. The conidia’s hydrophobic surface facilitates air dispersion and the spores can remain dormant and/or germinate in a wide range of environmental conditions [2]. *A. fumigatus* has a ubiquitous presence in the air that can reach up to 10^9^ conidia/m^3^ in certain environments [3]. Population genetic studies using simple sequence repeat (SSR) markers suggest that airborne dispersal by conidia has likely played a major role in the global population structure of *A. fumigatus* [4,5]. However, evidence for geographic specific SSR alleles and drug resistance profiles have also been reported, including for the Hamilton, Canada population of this species [6,7,8]. At present, whether the genetic uniqueness of geographic samples based on certain SSR markers reflects their whole-genome distinctiveness remains unknown. The main objectives of this study are to investigate the potential genetic uniqueness of the Hamilton population at the whole-genome sequence level and the potential relationship between genome sequence variation and susceptibility to the antifungal drug, amphotericin B (AMB).

*A. fumigatus* is among the most important opportunistic fungal pathogens in humans. Due to their high abundance in the air, conidia of *A. fumigatus* are inhaled by humans daily and are small enough (2 to 3 μm) to reach the lung alveoli. This can lead to a spectrum of fungal infections generally termed as aspergillosis. The disease can range from simple allergic reactions to severe invasive infections [1]. In immunocompetent individuals, inhaled conidia are cleared by the pulmonary immune system and rarely cause any harm. However, in immunocompromised individuals, incomplete killing of the fungi can lead to conidia germination followed by invasion of hyphae into tissue [2]. *A. fumigatus* is considered the primary cause of invasive aspergillosis, a life-threatening mold infection with high morbidity and mortality rates in immunosuppressed patients. Depending on factors such as patient population type, site of infection and treatment regimen, mortality rates associated with invasive aspergillosis range from 60 to 90 percent [3].

Among all antifungal agents, the triazole antifungals are usually first-choice drugs for treating aspergillosis because their use has been associated with better clinical response, lower infusion-related toxicity, lower nephrotoxicity, and increased survival [9]. However, the emergence of multiple-triazole resistant *A. fumigatus* strains throughout the world has been a growing public health concern and an increasing problem in treatment of patients in certain geographic areas. For cases of triazole-resistant *A. fumigatus* strains, amphotericin B (AMB) has been recommended by experts as core therapy [10].

AMB is a polyene antifungal agent that has been around since the 1950s for treatment against invasive fungal infections [11]. Although AMB has been in use for almost 70 years, its mechanism of action has not been completely elucidated. Instead, multiple mechanisms of action have been suggested over the years, such as the ion-channel model, the production of oxidative stress, the surface absorption model, and the sterol sponge model. The major benefits for AMB use are that it possesses a broad spectrum of action, being effective against most human pathogenic fungi, and that mycological resistance to AMB has been very uncommon. The antifungal is also fungicidal, in contrast to most azoles that are fungistatic. With the majority of affected patients being immunocompromised, the fungicidal effect of AMB is a desirable property in treatment and would be preferable for immunosuppressed patients where killing target fungal cells in a short period of time is needed. Indeed, resistance to AMB is less common than resistance to azoles across human fungal pathogens [12].

Recently, however, AMB resistance has been found in several *A. fumigatus* populations [13,14,15]. For example, the *A. fumigatus* population in Hamilton, Ontario, Canada was found to have a very high rate (96%) of AMB resistance [7]. Interestingly, also different from the majority of other geographic populations of *A. fumigatus*, the rate of triazole resistance in Hamilton was very low. Indeed, none of the 196 strains from three ecological niches (farm fields, city parks, and patients) was resistant to voriconazole or itraconazole, the two main triazole drugs used to treat *A. fumigatus* infections [8]. This high rate of AMB resistance in Hamilton was surprising because AMB has rarely been used in this region. Therefore, the observed AMB resistance in this and other regions may represent geographic-specific intrinsic resistance by the local populations. In addition, as AMB is the commonly recommended last-line drug of treatment and with the rising incidence of triazole resistance, the observed AMB resistance represents a major challenge for the healthcare system. At present, the mechanism(s) for the high rate of AMB resistance in Hamilton as well as other geographic regions are unknown.

Whole-genome sequencing (WGS) is a powerful approach to develop an understanding of population structure and antifungal resistance mechanisms in human fungal pathogens [16,17,18]. For example, WGS has been performed on *A. fumigatus* strains to investigate mutations causing azole resistance [19,20,21], however, a study focusing on AMB resistance in *A. fumigatus* has yet to be conducted. Indeed, there is little information about the mechanisms of AMB resistance in most human fungal pathogens. The aim of this study was to use a WGS approach to investigate genes associated with AMB resistance in *A. fumigatus*, with a focus on the Hamiltonian *A. fumigatus* isolates where the highest rate of AMB resistance has been reported. In addition, publicly available genome sequences of strains from a wide range of regions were included in our comparison to determine the relationships among strains from different geographic regions and to reveal the potential of the genetic uniqueness of the Hamiltonian *A. fumigatus* population at the genome level.

## 2. Materials and Methods

### 2.1. Isolates

This study included both our own strains from Hamilton, Canada and strains published previously by other groups. Our own strains included 12 strains, with 10 strains from Hamilton representing three ecological niches and a range of AMB susceptibilities. Five of the 10 strains were isolated from farmland soils around St. George (a township located ~35 km west of Hamilton), and three strains were obtained from urban park soils within Hamilton. The remaining two were clinical isolates obtained from the Clinical Microbiology Laboratory of Hamilton Health Sciences, located in the Hamilton General Hospital. None of the 10 *A. fumigatus* isolates from Hamilton are known to have been previously exposed to AMB. Each of the 10 strains have a different multilocus SSR genotype. As a comparison, we also included two model lab strains AFB62-1 and AFIR928, known as *A. fumigatus* supermaters. The details of all 12 strains are presented in Table 1. Information about the remaining 184 strains of *A. fumigatus* that we retrieved from the published literature where whole-genome sequences are available for comparison is presented in Supplemental Material Table S1.

### 2.2. Antifungal Susceptibility Testing

The in vitro susceptibility to AMB was tested for all 12 *A. fumigatus* strains. Testing was done following the M38-A2 guideline of the Clinical and Laboratory Standards Institute (CLSI) [22]. In brief, spores were grown on Sabouraud dextrose agar for 48 h at 37 °C. Conidial suspensions were adjusted to an optical density at 530 nm that ranged from 0.09 to 0.13. A 1:50 dilution was made in RPMI-1640 medium to obtain a final working concentration of approximately 0.4 × 10^5^ to 5 × 10^6^ CFU/mL. Spore suspensions were placed into the microtiter plates containing varying concentrations of AMB and incubated at 35 °C for 48 h. The concentrations tested were 0.25, 0.5, 1, 2, 4, 8, and 16 mg/L. *Candida parapsilosis* ATCC 22019 and *Candida krusei* ATCC 6258 were used as quality controls. The minimum inhibitory concentration (MIC) of AMB was determined as the lowest AMB concentration at which no colonies were observed by the naked eye. The CLSI epidemiological cut-off value of 2 mg/L was used to identify resistant strains [23].

### 2.3. DNA Extraction and Whole-Genome Sequencing

DNA extraction of Hamiltonian *A. fumigatus* isolates was carried out using a modified protocol described by Xu and colleagues [24]. Strains were grown on malt agar for 48 h at 37 °C. Conidia were then inoculated into 100 mL Sabouraud dextrose broth and incubated at 36 °C for 24 h with agitation at 200 rpm. Mycelia were filtered out using a sterile Whatman Grade 1 filter paper (Maidstone, UK), frozen with liquid nitrogen and ground to a powder with a pestle. The cells were resuspended in 0.5 mL protoplast buffer and incubated at 37 °C for 2 h. The suspensions were centrifuged at 5000 rpm for 10 min to collect protoplasts and remaining supernatant was discarded. 0.5 mL of lysing buffer was added in, followed by incubation at 65 °C for 30 min. 500 µL of chloroform/isoamyl alcohol (24:1) and 125 µL of 7.5 M ammonium acetate were added to the solution. The mixture was vortexed and centrifuged at 13,000 rpm for 15 min, or until the upper layer was clear. The clear upper layer was transferred into a new 1.5 mL tube, along with 550 µL of ice-cold isopropanol. The tube was mixed gently by inversion, centrifuged at 13,000 rpm for 2 min, and, remaining supernatant, discarded. DNA pellets were washed with 50 µl of 70% ethanol for 2 min, dried, and resuspended in 60 µL 1× TE buffer. The DNA was treated with 2 µL of 10 µg/mL RNase A at 30 °C for 1 h. Before storage at −20 °C, RNase A was removed from the samples with another round of chloroform/isoamyl alcohol extraction.

The DNA samples were sent to the Microbial Genome Sequencing Center in Pittsburgh, Pennsylvania, USA for whole-genome sequencing. Sequencing was conducted in a paired-end 2 × 15p mode on the Illumina Nextseq 550 platform at 50 times coverage.

### 2.4. Genome Sequences from NCBI

To identify the potential genomic uniqueness of the Hamilton *A. fumigatus* strains, we downloaded all published genome sequences of *A. fumigatus* from the NCBI Sequence Read Archive (SRA) as raw sequence data for comparison. This resulted in additional 184 *A. fumigatus* whole genomes sequences. Among these 184 strains, information on AMB susceptibility was available for 59 of the strains. Their detailed geographic origins, SRA accession numbers, and AMB susceptibilities for the 59 strains are presented in Table S1.

### 2.5. SNP Identification and Annotation

In this study, the focus of our genome sequence comparison is on single nucleotide polymorphisms (SNPs). To obtain the SNP profiles for each strain as compared to the reference strain Af293. Strain Af293 was chosen as the reference strain because it is a model lab strain for a variety of genetics study and because of its completeness in genome assembly and annotation to eight chromosomes. For all strains, the raw sequences (both downloaded from NCBI and our own) were first checked for quality using FastQC v0.11.5 [25]. Those of low quality were excluded using Trimmomatic v0.36 [26]. Reads were then mapped to the reference strain Af293 (GenBank accession GCA_000002655.1, with an AMB MIC of 1 mg/L [27]), using the BWA-MEM algorithm v0.7.17 [28]. Duplicate reads were marked using MarkDuplicates in the Picard tool [29] and variants were called using FreeBayes v0.9.21-19 [30]. SNP filtering and quality control were done using vcffilter in vcflib [31]. Low-quality calls with a quality score lower than 20 were filtered out. SNPs were concatenated, and invariant sites were removed. Variants were then annotated using SnpEff v5.0 [32] using the Af293 reference genome annotation to predict potential functional effects of SNPs.

### 2.6. SNP Distribution along Chromosomes

We analyzed SNP distributions along the eight chromosomes against the reference strain Af293 for each of the 71 strains with AMB susceptibility data. A total of 404,021 SNP sites were found among these strains and all were included for the analyses. For each strain, the SNP density along the chromosomes was calculated in 20 kb sliding windows shifted by 5 kb. The SNP densities were visualized with R package plotly v4.9.2.1 [33].

### 2.7. Phylogenetic Analysis

A phylogenetic tree was constructed for all 196 *A. fumigatus* genomes using RAxML v8.0.25 [34], with 100 bootstrap replicates and rooted to the reference strain Af293. Information for all 404,021 SNP sites was used to construct the strain relationships. The tree was then visualized using iTOL v4 [35].

Cluster analysis was also conducted on the 71 strains with known AMB susceptibilities. In this subset of analyses, only SNPs in gene coding regions among the 71 strains were included, with a total of 117,770 sites included in the analyses. For this analysis, SNP genotype data were first re-coded as 0, 1, 2, and 3 for reference and different alternative genotypes to construct a matrix. To identify the optimal number of clusters, the gap statistic was computed using the function “clusGap” in R software, with the maximum number of clusters set to 8 genetic clusters and with 50 bootstraps. The number of 8 genetic clusters was chosen here based on an earlier estimate of the global population of over 2000 isolates using 9 SSR genetic markers. K-means clustering was performed on the matrix using R package stats v3.5.1 [36].

### 2.8. Genome-Wide Association Study

We used PLINK v1.9 [37] to identify if specific SNPs are significantly associated with AMB susceptibility. A linear mixed model was used, and AMB MIC data were treated as a quantitative trait. To alleviate the influence of different allele frequencies from different genetic populations, only samples from Cluster 2 (*n* = 34) were included in the association analysis. Among these samples, 15 had AMB MIC values ≥ 2 mg/L. For quality control (QC), SNPs with a minor allele frequency lower than 0.05 were removed. Cryptic related individuals with pihat above 0.2 were also excluded from the analysis. After the QC processing, 136,064 variants and 34 samples remained. To reduce the influence of significantly linked SNPs in the association analysis, r2 was computed between pairs of SNPs with a sliding window of size 50, which was shifted using size 5. Pairs of SNPs with r^2^ higher than 0.2 were pruned out. After pruning, 94,703 of the 136,064 SNPs were removed. Furthermore, to reduce the effect of population structuring on the association among SNPs, the principal components for the remaining 41,361 SNPs were computed. The top 10 principal components were included in the linear regression model as covariates.

### 2.9. Data Availability

Genome sequence accession numbers for the 12 isolates that we sequenced for this study are provided in Table 1. Accession numbers for the 184 strains downloaded from the NCBI SRA are listed in Supplemental Material Table S1.

## 3. Results

### 3.1. AMB Susceptibility

Table 1 summarizes AMB MIC results for our 12 *A. fumigatus* strains sent for whole-genome sequencing. CON4 was susceptible to AMB, with an MIC of 1 mg/L, and CM21 had an intermediate resistance at 2 mg/L. The remaining 10 isolates were all AMB resistant with MICs greater than 2 mg/L. The isolate CM11 had the highest MIC of 8 mg/L. The allelic information at nine simple sequence repeat loci commonly used for genotyping strains of *A. fumigatus* are also presented for each of the 12 strains in Table 1.

Aside from our 12 isolates with AMB susceptibility data, 59 additional *A. fumigatus* genomes with reported AMB MIC values were obtained from NCBI SRA (Table S1). The AMB MIC values for the 59 isolates are listed in Table 2, grouped by geographic location. Of the 59 additional strains, 5 (8.47%) had AMB MICs of 2 mg/L. All five of these strains were obtained from the Chiba University Hospital in Japan. The remaining 54 isolates were all susceptible to AMB, with MIC values below 2 mg/L (Table 2).

### 3.2. SNP Distribution along Chromosomes

**Figure 1:**
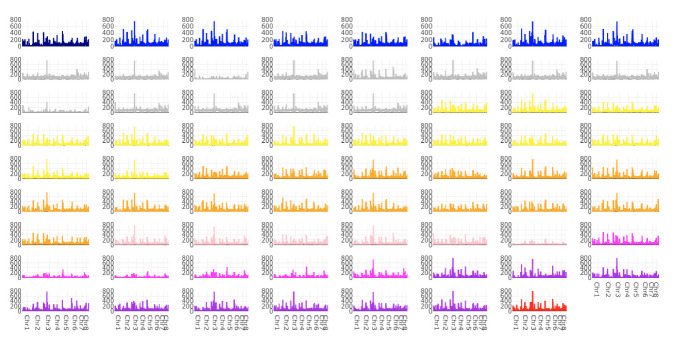
Single nucleotide polymorphism (SNP) density plots across eight chromosomes representing the number of SNPs within 20 kb window size. The color indicates the strain’s AMB MIC value: 0.06 (navy), 0.125 (blue), 0.1875 (grey), 0.25 (yellow), 0.5 (orange), 1 (light pink), 2 (pink), 4 (purple) and 8 (red) mg/L.

A total of 404,021 SNPs were identified in our sample of 196 isolates. Genome-wide SNP density plots were constructed for each of the 71 isolates with known AMB susceptibilities when compared to the reference strain Af293, including all 404,021 SNPs. SNPs were distributed across the eight chromosomes in each genome (Figure 1). Chromosome 3 showed the most SNPs. However, within each chromosome, there was significant heterogeneity in SNP distribution among chromosomal segments when each of the strain was compared to the reference genome Af293. We note here three SNP-rich regions, each region with over 500 SNPs observed: in the 25 kb region from 4805 kb to 4830 kb of chromosome 1, the 20 kb region from 1010 kb to 1030 kb of chromosome 3, and the 20 kb region from 95 kb to 115 kb of chromosome 5. Overall, we did not see a consistent pattern of large-scale SNP distribution differences among strains with different AMB susceptibilities.

### 3.3. Phylogenetic Analysis

**Figure 2:**
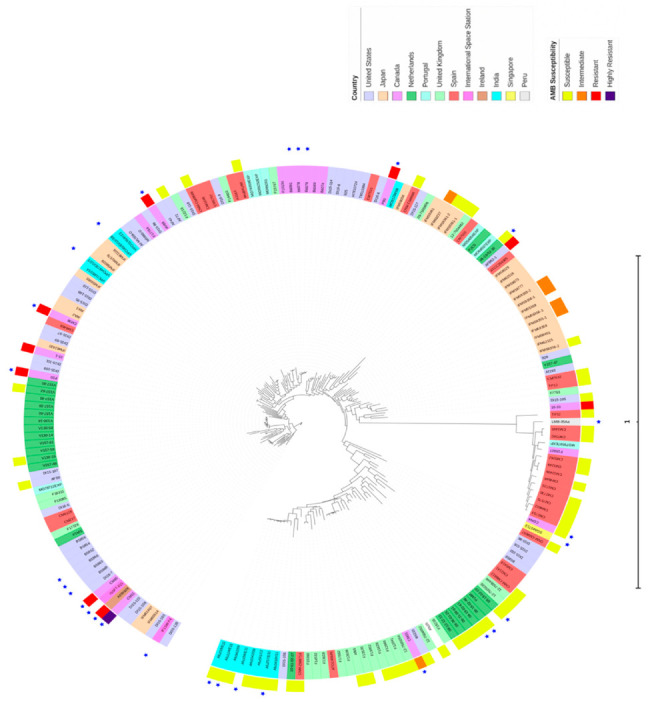
Phylogenetic tree of the 196 *A. fumigatus* strains in the study. Circular phylogram of maximum-likelihood phylogeny rooted to the reference Af293 was constructed using the Randomized Axelerated Maximum Likelihood (RAxML) and Interactive Tree Of Life (iToL) programs. Scale bar represents substitutions per nucleotide site.

We compared the 10 Hamilton isolates to the remaining 186 *A. fumigatus* genomes, which were obtained from 11 different countries, by generating a maximum-likelihood phylogenetic tree using all 404,021 SNP sites (Figure 2). In this figure, the outer-most label showed their ecological source, with the blue star representing strains from the environment, while the remaining ones were from clinical sources. Though several environmental strains were clustered, overall, our data showed that clinical and environmental strains were interspersed among each other on the phylogenetic tree. The second circle from outside represented AMB susceptibilities, with yellow representing susceptible, orange representing intermediate-resistant, red representing resistant, and purple representing highly resistant strains. The third circle from outside contained strain names highlighted in different colors representing different countries of origins. The branch lengths shown in the inner phylogeny were proportional to strain genetic distances. Our analyses identified that these 196 strains belonged to three distinct clusters. Both clinical and environmental strains were found in all three clusters. One cluster (Cluster 1) contained 15 strains, including 12 strains from Spain (CM2733, CM4602, CM7570, CM2730, CM3720, CM4946, CM3249b, CM3249, CM3262, CM7560, CM2495 and TP32), 1 strain from Canada (F15927), 1 strain from India (MO79587EXP), and 1 strain from Peru (LMB-35Aa). Cluster 1 strains were genetically highly divergent from the remaining isolates. All isolates in Cluster 1 tested for AMB susceptibility were susceptible to AMB. The second cluster contained 133 strains, and they were from diverse geographic regions. Almost all AMB resistant isolates among the sequenced strains were in Cluster 2. The remaining 48 strains formed the third cluster and these 48 strains were also from broad geographic regions. Among the 29 strains in this cluster with AMB susceptibility data, only one from Hamilton showed AMB resistance, with the remaining 28 being susceptible to AMB.

**Figure 3:**
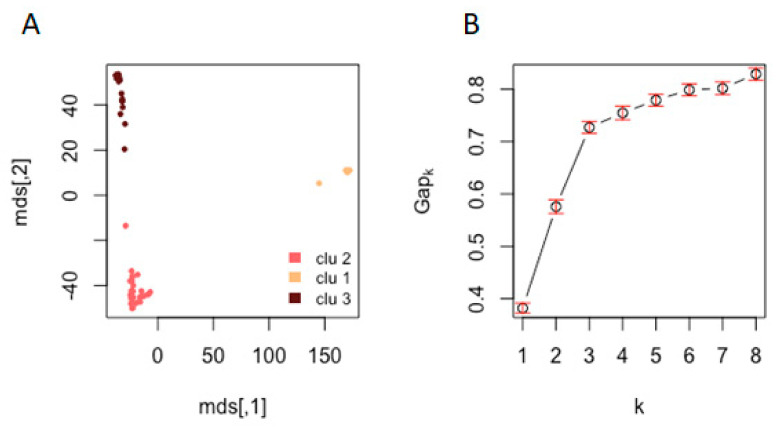
(**A**) Multidimensional scaling (MDS) plot of SNP profiles. (**B**) Gap curve where x-axis is the number of clusters and y-axis is the gap statistic. The MDS plot of 71 isolates (and Af293) and the gap curve show that samples could be clustered into three populations.

Cluster analysis was further conducted on the 71 strains with known AMB susceptibilities using SNPs in protein-coding regions. Their comparisons with the reference strain Af293 identified 117,770 sites in the CDS regions in the 71 samples. The results showed that our strains were divided into 3 clusters (Figure 3A), similar to results based on all 196 strains. In this analysis, although the highest Gapk value was when k = 8, the changes in Gapk were relatively minor after k = 3 (Figure 3B), consistent with the multidimensional scaling results of three genetic clusters shown in Figure 3A. Our subsequent analyses between SNP and AMB susceptibility associations among the 71 strains were based on k = 3.

Among the three clusters, Cluster 2 consisted of 33 strains with AMB susceptibility data, including the reference Af293. In Cluster 2, 15 (45.45%) strains were AMB-resistant (Table 3). Cluster 3 consisted of 28 strains, with only 1 (3.57%) strain being AMB resistant (Table 3). Meanwhile, all 10 strains from Cluster 1 were susceptible to AMB (Table 3). Fisher’s exact test (one-tailed) showed that different clusters had significantly different proportions of AMB resistant strains (*p*-value = 0.0001). Indeed, among the 10 Hamilton strains, 9 were found in Cluster 2 and the remaining 1 strain belonged to Cluster 3 (Table 3). The only strain in Cluster 3 showing resistance to AMB was from Hamilton, Canada, strain CM21.

### 3.4. Genes of Interest

In this analysis, we retrieved all the genes related to ergosterol biosynthesis and redox homeostasis from the *A. fumigatus* genomes. Their SNP profiles were then determined for each of the 71 isolates at these genes. A total of 22 genes were analyzed, including six in the ergosterol biosynthesis pathway, seven in the reactive oxygen species (ROS)-detoxifying systems, and nine in the high-osmolarity glycerol mitogen-activated protein kinase (HOG MAPK) pathway (Table 4). These three pathways have been suspected as involved in AMB susceptibility differences in fungi. Here, our focus is on the distributions of specific SNPs among the various groups of strains with different AMB susceptibilities, in an attempt to identify the specific mutations associated with AMB resistance.

Specifically, we examined all high-quality non-synonymous SNPs in genes *ERG2, ERG3, ERG4, ERG6, ERG11* and *ERG13* of the ergosterol biosynthesis pathway; in genes *CatA, Cat1, Cat2, Sod1, Sod2, Sod3* and *Sod4* of the reactive oxygen species (ROS) detoxification pathway; and in genes S*kn7, Fos1, TcsB, Pbs2, Sho1, SakA, MpkA, MpkB* and *MpkC* of the high-osmolarity glycerol (HOG) mitogen-activated protein kinase (MAPK) signaling pathway (Table S2) for their potential associations with AMB susceptibility. For each non-synonymous SNP, the association between genotype and phenotype (in this case, AMB resistance) was measured using Fisher’s exact test (one-tailed), with the significance level set to 0.05. There were 12 SNPs with *p*-values below this threshold, and they were considered as statistically significant (Table 5). These SNPs were in *ERG3* (*n* = 2), *TcsB* (*n* = 4), *MpkC* (*n* = 2), *CatA* (*n* = 2), *Fos1* (*n* = 1) and *MpkB* (*n* = 1). With Af293 genome as the reference, the four SNPs located in *TcsB* were associated with increased susceptibility to AMB, while the remaining eight SNPs were associated with increased resistance to AMB.

### 3.5. Genome-Wide Association Analysis

**Figure 4:**
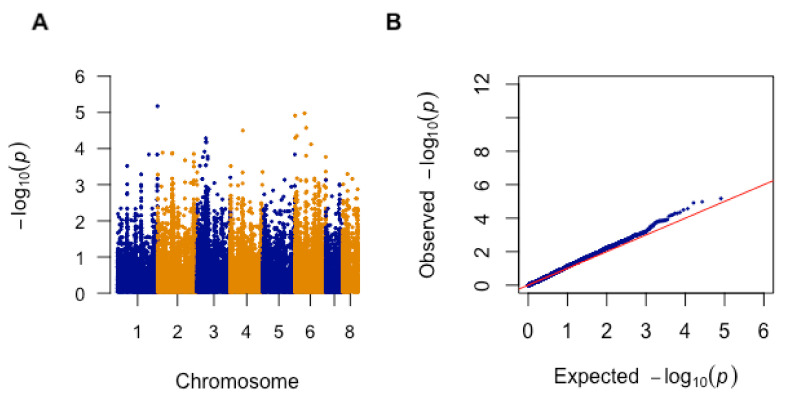
(**A**) The Manhattan plot for AMB sensitivity of *A. fumigatus isolates* in Cluster 2 (*n* = 33). The plot is based on −log_10_(*p*-value) and chromosome position. (**B**) Quantile–quantile (Q–Q) plot from genome-wide association (GWA) analysis of AMB sensitivity. Comparison of observed −log_10_(*p*-value) to the expected −log_10_(*p*-value).

Aside from the targeted analyses of the 22 genes in the three pathways mentioned above, we also performed a genome-wide association study (GWAS) between SNPs and AMB susceptibility for strains in Cluster 2. The results are summarized in Figure 4A as a Manhattan plot (Figure 4A). A quantile–quantile (Q–Q) plot of observed versus expected −log_10_(*p*-value) was also generated, which revealed there was no inflation of false positive results (Figure 4B).

From the GWAS result, 15 representative SNPs with a LOD score of greater than 3.86 are shown in Table 6. Among the 15 SNPs, 13 (86.67%) were located in intergenic regions and two (13.33%) were found within coding regions (Table 6). These two SNPs were mapped to chromosome 6 in the gene *Afu6g04940* and to chromosome 3 in *Afu3g03350*, respectively. Of these two SNPs, that on *Afu3g03350* was a nonsynonymous mutation, from glutamine to lysine at amino acid position 1552.

**Figure 5:**
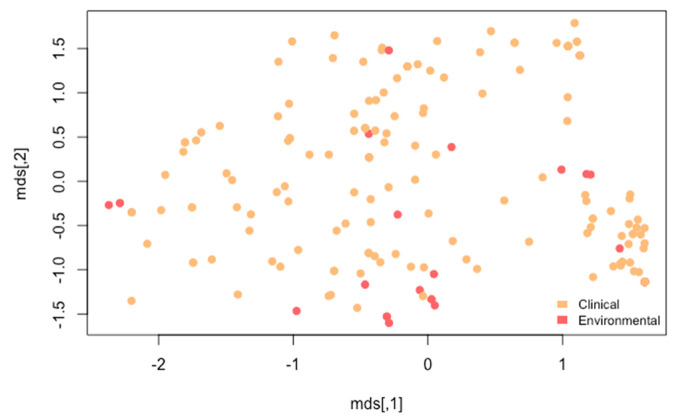
Multidimensional scaling (MDS) plot showing relationships among 196 strains based on the top 65 SNPs that showed associations with AMB susceptibilities. Each filled circle represents a strain. Circles in red were of environmental origins, while those in brown were from clinical sources.

We also looked at all SNPs within CDS regions and have listed the top 65 with the lowest *p*-values in GWAS in Table S3. Based on these 65 SNPs, we analyzed whether environmental strains and clinical strains grouped differently. Our results indicated that the two groups of strains were dispersed among each other (Figure 5). This result is similar to that shown in Figure 2. However, these 65 SNPs did not separate the 196 strains into three distinct genetic clusters as shown in Figure 2 (based on the whole-genome SNP profiles) and Figure 3A (based on SNPs in all CDS among 71 strains with AMB susceptibility data).

## 4. Discussion

In this study, we investigated the overall phylogenomic diversity of *A. fumigatus* using whole-genome sequences of 196 strains collected from 11 different countries: Canada, India, Ireland, Japan, Netherlands, Peru, Portugal, Singapore, Spain, United Kingdom and the United States. Using 404,021 SNP sites, our phylogenetic analysis identified a group of strains that had a high level of divergence from all other *A. fumigatus* strains. This clade consisted of strains from Spain (*n* = 12), Canada (*n* = 1), India (*n* = 1) and Peru (*n* = 1). Our results are consistent with what had been reported by Garcia-Rubio and colleagues [38]. Their study examined 101 *A. fumigatus* genome sequences from seven countries (Canada, India, Japan, Netherlands, Portugal, Spain, and the United Kingdom) and included the same strains from Spain (*n* = 12) and Canada (*n* = 1). They conducted a phylogenetic analysis using all 101 genomes. The result was that these 13 isolates clustered together and were identified as belonging to the most distant cluster. The cluster was also found to have the greatest homogeneity, with differences between strains being the lowest of all other groupings [38]. Our analyses further extended their analyses for this clade and expanded its geographic distribution into South America (Peru) and Asia (India). The significant divergence of strains in this cluster from other strains suggest that Cluster 1 strains likely represent a cryptic species within the A. fumigatus sensu stricto complex. In addition, the relatively limited sequence different among geographically distant strains in this cluster suggests recent dispersals of Cluster 1 strains among geographic regions.

Our analyses focused on the 71 strains with known AMB susceptibilities to investigate potential genomic regions associated with AMB resistance. The 71 strains were obtained from 8 countries: Canada, India, Ireland, Japan, Netherlands, Spain, United Kingdom, and the United States. Clustering analysis was conducted using 117,770 SNP sites from CDS regions, and it showed the existence of three genetic clusters, consistent with the results based on all SNPs from the 196 genomes. However, our results are slightly different from that based on 101 genomes by Garcia-Rubio and colleagues [38]. They determined that their *A. fumigatus* samples were divided into four clusters, while our analyses showed three clusters. This difference in cluster number was because our Cluster 2 was a combination of their Clusters I and III. This combination was likely due to the addition of 95 genomes representing more geographic regions which could have resulted in intermediates that linked these two clusters (Clusters I and III) together to form one larger cluster (our Cluster 2). In addition, our clustering result showed a noticeably lower number of genetic clusters from what was reported earlier based on SSR markers e.g., [4]. For example, in the study by Ashu et al. [4], they analyzed the population structure of 2025 *A. fumigatus* isolates from 13 countries (Australia, Belgium, China, Cuba, France, Germany, India, Italy, Netherlands, Norway, Spain, Switzerland and the United States) using nine SSR markers. Although their study analyzed more strains (2025) than this study (196 strains), the number of markers used in the current study (404,021 SNPs for the whole sample of 196 strains and 117,770 SNPs for the 71 strains) is much greater than the nine microsatellite loci. Nonetheless, the Ashu et al. [4] study had samples from 13 countries (vs. 11 countries in this study) and included strains from 9 countries that we did not have in our study, which may account for some of the discrepancy.

Our study also examined non-synonymous SNPs in 22 genes of interest and the *p*-values for each SNP were calculated using Fisher’s exact test. We found 12 missense mutations that were significantly associated with AMB resistance. The SNPs were located in six genes, *CatA, ERG3, Fos1, MpkB, MpkC*, and *TcsB*. These 22 genes were chosen based on the known mechanisms of AMB resistance in other fungi [39]. Previous studies have found a correlation between alterations in the ergosterol synthesis pathway and AMB resistance in several fungal species. Here, we found two SNPs in *ERG3* that were significantly associated with AMB resistance. *ERG3* is involved in the ergosterol biosynthesis pathway and encodes for C-5 sterol desaturase. The role of *ERG3* in AMB resistance has also been validated in AMB resistant *Candida lusitaniae* strains where *ERG3* expression was found to be reduced [40]. Additionally, other studies have found an association between oxidative stress tolerance and AMB resistance. Therefore, we also examined genes involved in the two ROS-detoxifying systems, specifically catalases and superoxide dismutases. Moreover, in a previous study on *Candida dubliniensis* and *Candida albicans*, superoxide dismutase and catalase activities were significantly higher in AMB resistant strains [41]. There are three functional catalases in *A. fumigatus*: *Cat1, Cat2* and *CatA*. Our results showed that two SNPs in *CatA* were significantly associated with resistance. The final category we looked at were genes involved in the HOG MAPK signaling pathway, as the pathway plays a role in oxidative stress response in many fungal pathogens. This pathway has also been observed to regulate genes involved in ergosterol biosynthesis. For example, inhibition of the HOG pathway increased the expression levels of *ERG11* in *Cryptococcus neoformans* [42]. Our analyses found four SNPs in *TcsB* to be associated with AMB sensitivity. SNPs associated with AMB resistance were also found in *Fos1* (*n* = 1), *MpkB* (*n* = 2) and *MpkC* (*n* = 2). To our knowledge, these four genes have not been implicated in AMB resistance, and they represent promising candidates for further analyses.

To investigate AMB resistance in *A. fumigatus* on a broader scale, whole-genome sequences of the 71 strains were used in GWAS. Of the top 15 SNPs significantly associated with AMB MIC, two significant SNPs in genic regions were identified: a synonymous variant in *Afu6g04940* and a missense variant in *Afu3g03350*. *Afu6g04940* is annotated as the cytokinesis protein SepA/Bni1. Although not much is known about its role in *A. fumigatus*, its ortholog has been studied in the closely related model species *Aspergillus nidulans*. The *A. nidulans* ortholog, *SepA*, is involved in polarized growth, specifically apical growth and septation [43]. *SepA* deletion mutants are unable to undergo septation due to their inability to assemble actin rings at division sites [44]. The mutants still form hyphae, but they are wider and have an abnormal dichotomous branching pattern due to defects in hyphal polarity [44]. The second discovered gene, *Afu3g03350* or *SidE*, encodes a bimodular peptide synthetase [45]. *SidE* was previously thought to be involved in siderophore production but is now found to be involved in fumarylalanine (FA) production [45]. Although the biological function of fumarylalanine remains unknown, due to the structural similarity of FA to established pharmaceuticals with immunomodulatory activity, the gene may be involved in host immunosuppression and *A. fumigatus* virulence [45]. Expression of *SidE* was also found to be significantly transcriptionally upregulated at increased temperatures, under both presence and absence of iron, as well as by oxidative stress in the presence of iron (in iron-replete or high-iron conditions) [45].

At present, the origin of the high rate of AMB resistance in Hamilton, Canada and several other countries remains unknown. However, the results of our study have brought into light several possibilities. In the first, the genes identified here as related to AMB resistance, including *Afu6g04940* and *Afu3g03350,* suggest the possibility that AMB resistance could be related to iron and/or temperature conditions in the environment, where mutations that occurred in response to these stresses led to increased AMB tolerance as a by-product. External cell wall stress has also been found to induce alternative cytokinesis and septation strategies in fungal species. Additionally, iron acquisition and the oxidative stress response are known to be related, with iron shown to play an essential role in the oxidative stress response [46]. The second possibility is related to mutational rates. Several studies have suggested that environmental stress could increase the mutational rate [47]. It is thought that the maintenance and repair pathways function at a lower capacity during stress, which in turn leads to more mutations [47]. This would ultimately result in a higher chance for the appearance of mutations that confer resistance [47]. The high allelic and genotypic diversity of the Hamilton strains at both the SNP and microsatellite loci are consistent with the high mutation rates in this population. Finally, the Hamilton strains were found mainly in Cluster 2 (90%), which could suggest a potential evolutionary predisposition of this groups of strains being more likely to develop AMB resistance. These three possibilities are not mutually exclusive of each other and all could contribute to the observed high prevalence of AMB resistance. Indeed, the close relationships among clinical and environmental strains at the genome level as shown in both Figure 2 and Figure 5 suggest that selection pressure on environmental strains of *A. fumigatus* can have significant impacts on clinical practices on antifungal drug treatments.

The incidence of invasive aspergillosis has been increasing, due in part to the expanding immunocompromised population. Invasive aspergillosis is becoming an important infectious disease, with *A. fumigatus* accounting for the majority of cases. Over the past decade, incidence of invasive aspergillosis in low-risk patients and immunocompetent individuals have also been increasingly reported [48,49,50]. Therefore, antifungal susceptibility patterns and information on resistance mechanisms is becoming more important. Understanding the underlying mechanisms leading to the origin and persistence of AMB-resistant strains could help us developing measures to reduce their emergence and spread. Furthermore, while our current knowledge on AMB resistance in *A. fumigatus* is still fragmentary, GWAS analysis in this study identified a large number of SNPs significantly associated with AMB resistance. We highlighted the significant SNPs associated with AMB susceptibility in eight genes, *Afu6g04940*, *Afu3g03350, ERG3, Fos1, CatA, TcsB, MpkB*, and *MpkC*. The role of these eight genes as well as other genes in AMB resistance still need to be confirmed through additional experiments and functional assessments. Nonetheless, the results obtained from this study provide a foundation for further research on AMB resistance. From a clinical perspective, the results could aid in the development of diagnostic molecular markers for AMB resistance screening. This information would help predict the outcome of AMB therapy and determine whether it remains the best option as the last line drug in treatment. With prompt treatment being crucial in cases of invasive aspergillosis, the potential diagnostic markers to be developed in this study could aid in and speed up clinical decision making.

## Figures and Tables

**Table 1:** Microsatellite genotypes of 12 *Aspergillus fumigatus* strains and their in vitro susceptibility to amphotericin B.

Samples	BioSample Accession Number	City, Country	Alleles at the Nine Consensus SSR Loci									AMB MIC (mg/L)
			2A	2B	2C	3A	3B	3C	4A	4B	4C	
CON4	SAMN16550813	Hamilton, Canada	21	13	11	5	24	46	11	9	10	1
CM21	SAMN16550814	Hamilton, Canada	6	25	8	32	5	6	23	8	9	2
CM65	SAMN16550815	Hamilton, Canada	8	26	10	15	20	43	7	7	8	4
P80	SAMN16550816	Hamilton, Canada	13	31	15	10	10	33	8	8	9	4
15-1	SAMN16550817	Hamilton, Canada	14	33	16	36	16	12	5	5	6	4
AV88	SAMN16550818	Hamilton, Canada	6	24	8	15	9	30	8	7	8	4
15-33	SAMN16550819	Hamilton, Canada	10	28	12	29	7	29	12	8	9	4
P20	SAMN16550820	Hamilton, Canada	11	29	13	10	28	21	15	7	8	4
CM38	SAMN16550821	Hamilton, Canada	10	28	11	27	27	49	8	6	7	4
CM11	SAMN16550822	Hamilton, Canada	15	33	17	11	31	53	7	7	8	8
AFB62-1	SAMN16550823	San Antonio, United States	16	14	11	23	16	24	4	10	N/A	4
AFIR928	SAMN16550824	Dublin, Ireland	16	10	14	24	10	21	7	7	4	4

**Table 2:** In vitro amphotericin B (AMB) susceptibility profiles of 59 *A. fumigatus* isolates from the published reports, grouped by their country origins.

Country	Number of Isolates	AMB MIC (mg/L)			
		≤0.25	0.5	1	2
United States	4	2	2	0	0
United Kingdom	11	6	5	0	0
Netherlands	10	4	6	0	0
India	7	6	1	0	0
Spain	18	16	1	1	0
Japan	8	0	0	3	5
Unknown ^a^	1	0	0	1	0
Total	59	34	15	5	5

^a^ The geographical location for isolate Afs35 is unknown.

**Table 3:** K-means cluster analysis results for strains with AMB susceptibility data. Isolate names and their geographic origins for all 71 strains are also shown in this table.

Cluster Group	Isolate	Country	AMB MIC (mg/L)
1	CM2495	Spain	0.1875
1	CM2730	Spain	0.1875
1	CM2733	Spain	0.1875
1	CM3249	Spain	0.1875
1	CM3262	Spain	0.1875
1	CM4602	Spain	0.1875
1	CM4946	Spain	0.1875
1	CM7560	Spain	0.1875
1	CM7570	Spain	0.1875
1	TP32	Spain	0.1875
2	15-1	Canada	4
2	15-33	Canada	4
2	AV88	Canada	4
2	CM11	Canada	8
2	CM38	Canada	4
2	CM65	Canada	4
2	CON4	Canada	1
2	P20	Canada	4
2	P80	Canada	4
2	AFIR928	Ireland	4
2	IFM59355-1	Japan	2
2	IFM59355-2	Japan	2
2	IFM59356-1	Japan	2
2	IFM59356-2	Japan	1
2	IFM59356-3	Japan	2
2	IFM59361-1	Japan	1
2	IFM59361-2	Japan	1
2	IFM60237	Japan	2
2	08-19-02-30	Netherlands	0.5
2	V130-15	Netherlands	0.5
2	V157-62	Netherlands	0.25
2	akuBKU80	Spain	0.1875
2	CM7632	Spain	0.1875
2	CNM-CM8057	Spain	0.25
2	CNM-CM8686	Spain	0.5
2	CNM-CM8689	Spain	1
2	TP12	Spain	0.1875
2	09-7500806	United Kingdom	0.5
2	12-7504462	United Kingdom	0.5
2	AF72	United States	0.25
2	AF90	United States	0.5
2	AFB62-1	United States	4
2	DI15-105	United States	0.125
3	CM21	Canada	2
3	Afu1042/09	India	0.25
3	Afu124/E11	India	0.125
3	Afu218/E11	India	0.125
3	Afu257/E11	India	0.125
3	Afu343/P/11	India	0.125
3	Afu591/12	India	0.5
3	Afu942/09	India	0.25
3	08-12-12-13	Netherlands	0.25
3	08-19-02-10	Netherlands	0.25
3	08-19-02-46	Netherlands	0.5
3	08-19-02-61	Netherlands	0.25
3	08-31-08-91	Netherlands	0.5
3	08-36-03-25	Netherlands	0.5
3	10-01-02-27	Netherlands	0.5
3	CNM-CM8714	Spain	0.25
3	CNM-CM8812	Spain	0.25
3	12-7504652	United Kingdom	0.25
3	12-7505054	United Kingdom	0.5
3	12-7505220	United Kingdom	0.5
3	12-7505446	United Kingdom	0.25
3	Af65	United Kingdom	0.5
3	F11628	United Kingdom	0.06
3	F12041	United Kingdom	0.25
3	F12865	United Kingdom	0.125
3	F13535	United Kingdom	0.125
3	Afs35	Unknown	1
3	B5233	United States	0.5

**Table 4:** Selected genes of interest involved in ergosterol biosynthesis and redox homeostasis.

Category	Gene	Protein Description
Ergosterol biosynthesis	*ERG11*	14-alpha demethylase
	*ERG3*	Sterol delta 5,6-desaturase
	*ERG4*	C-24(28) sterol reductase
	*ERG6*	Sterol 24-C-methyltransferase
	*ERG13*	Hydroxymethyl glutaryl-coenzyme A synthase
	*ERG2*	C-8 sterol isomerase
ROS ^a^-detoxifying systems	*CatA*	Spore-specific catalase
	*Cat1*	Mycelial catalase
	*Cat2*	Putative bifunctional catalase-peroxidase
	*Sod1*	Copper-zinc superoxide dismutase
	*Sod2*	Manganese-superoxide dismutase
	*Sod3*	Manganese superoxide dismutase
	*Sod4*	Copper-zinc superoxide dismutase
HOG MAPK ^a^ pathway	*Skn7*	Transcription factor and response regulator
	*Fos1*	Histidine kinase
	*TcsB*	Sensor histidine kinase
	*Pbs2*	Mitogen-activated protein kinase kinase
	*Sho1*	Transmembrane osmosensor
	*SakA*	Mitogen-activated protein kinase
	*MpkA*	Mitogen-activated protein kinase
	*MpkB*	Mitogen-activated protein kinase
	*MpkC*	Mitogen-activated protein kinase

^a^ ROS = reactive oxygen species; HOG MAPK = high-osmolarity glycerol mitogen-activated protein kinase.

**Table 5:** Non-synonymous SNPs located in six of the 22 genes of interest that are significantly associated with AMB resistance/sensitivity based on Fisher’s Exact test (*p* < 0.05).

Chromosome	Position	Gene	Amino Acid Change	Predicted Effect	*p*-Value (Fisher’s Test)
2	61,543	*ERG3*	Threonine to Isoleucine	Missense Variant	0.01254
2	62,002	*ERG3*	Tyrosine to Phenylalanine	Missense Variant	0.01254
2	145,934	*TcsB*	Aspartic acid to Glycine	Missense Variant	0.00081
2	146,469	*TcsB*	Glycine to Serine	Missense Variant	0.00207
2	147,363	*TcsB*	Arginine to Glycine	Missense Variant	0.00081
2	147,396	*TcsB*	Alanine to Proline	Missense Variant	0.00013
5	2,342,264	*MpkC*	Tryptophan to Serine	Missense Variant	0.00029
5	2,342,466	*MpkC*	Isoleucine to Threonine	Missense Variant	0.00003
6	857,963	*CatA*	Aspartic acid to Asparagine	Missense Variant	0.01195
6	858,366	*CatA*	Serine to Asparagine	Missense Variant	0.00007
6	2,533,399	*Fos1*	Alanine to Aspartic acid	Missense Variant	0.04172
6	3,232,955	*MpkB*	Lysine to Arginine	Missense Variant	0.00084

**Table 6:** Top 15 significant SNPs associated with AMB resistance based on a genome-wide association study (GWAS).

Chromosome	SNP ID	Position (bp)	Reference	Alternative	−log10(*p*-value)	Gene ID	Predicted Effect
1	59037	4879185	C	T	5.17	Afu1g17700-Afu1g17710	Intergenic Region
6	304875	1168808	A	C	4.98	Afu6g04940	Synonymous Variant
6	288764	25934	C	T	4.91	Start of Chr.6-Afu6g00110	Intergenic Region
6	308832	1355924	C	G	4.57	Afu6g06350-Afu6g06360	Intergenic Region
4	208608	1472947	T	C	4.50	Afu4g04820-Afu4g05830	Intergenic Region
6	294968	224108	G	A	4.35	Afu6g00770-Afu6g01790	Intergenic Region
6	288893	26965	G	A	4.29	Start of Chr.6-Afu6g00110	Intergenic Region
3	144422	1046030	A	G	4.29	Afu3g03760-Afu3g03780	Intergenic Region
3	147694	1070350	T	C	4.19	Afu3g03760-Afu3g03780	Intergenic Region
3	150064	1085944	A	G	4.17	Afu3g03760-Afu3g03780	Intergenic Region
6	315265	1967652	A	C	4.12	Afu6g08400-Afu6g08410	Intergenic Region
3	139709	897091	C	A	3.92	Afu3g03350	Missense Variant
2	68354	586421	A	T	3.89	Afu2g023300-Afu2g02340	Intergenic Region
2	81978	1783765	CAGGGC	TAGGGC, CAGGGT	3.88	Afu2g06205-Afu2g06220	Intergenic Region
2	80580	1773933	T	C	3.86	Afu2g06205-Afu2g06220	Intergenic Region

## Supplementary Materials

The following are available online at https://www.mdpi.com/2076-2607/8/11/1673/s1. Table S1: Information on the *Aspergillus fumigatus* genomes obtained from the NCBI Sequence Read Archive. Table S2: All identified SNPs in the 22 genes of interest for the 71 *A. fumigatus* strains. Table S3: Top 65 associated SNPs in CDS regions identified through GWAS analysis.
